# Supplementary material for: Water orientation and dynamics in the closed and open influenza B virus M2 proton channels
Source: Commun Biol. 2021 Mar 12;4:338. doi: 10.1038/s42003-021-01847-2 (PMC7955094; doi:10.1038/s42003-021-01847-2)
Supplement: Supplementary file 1 — Supplementary Information [file 42003_2021_1847_MOESM1_ESM.pdf]

Supplementary Information

Water Orientation and Dynamics in the Closed and Open Influenza B Virus M2 Proton Channels

Martin D. Gelenter[$], Venkata S. Mandala[$], Michiel J.M. Niesen[$], Dina A. Sharon[$], Aurelio J. Dregni, Adam P. Willard, and Mei Hong*

Department of Chemistry, Massachusetts Institute of Technology, 77 Massachusetts Avenue, Cambridge, MA 02139

[$] These authors contributed equally
* Corresponding author: Mei Hong: meihong@mit.edu

Supplementary Methods
Water-protein spin diffusion data analysis

Here we derive the analytical equations for the square-root time dependence of the buildup intensities of water transfer to protein. We also prove how the relative intensities of the initial-regime water transferred intensities reflect the relative amounts of water.

Spin diffusion from a one-dimensional point source can be described analytically[1] as:

$$\mathrm{M}(x,t) = \frac{W}{\sqrt{\pi D t}} \exp\left(\frac{-(x - x_S)^2}{4Dt} \right) \tag{1}$$

where M is the magnetization at position *x* and time *t*, x_S is the position of the point source, *W* is the amount of water contributing to diffusion, and *D* is the effective spin diffusion coefficient for magnetization transfer between water and protein, which has contributions from both chemical exchange and spin diffusion [2]. We assume that the spin diffusion coefficients of water in the high- and low-pH channels is approximately the same and is much greater than the effective diffusion coefficient D. Since proton chemical exchange is faster at pH 7.5 than at pH 4.5 [3-5], $D_{pH7.5}$ is greater than or equal to $D_{pH4.5}$. Thus W is the quantity of interest for the water-count experiments. In our samples, magnetization transfer originates from a continuum of water molecules at different positions to protein. This modifies the diffusion equation to:

$$\mathrm{M}_{\mathrm{dom}}(x,t) = \int_{-\infty}^{0} \frac{W}{\sqrt{\pi D t}} \exp\left(\frac{-(x - x_S)^2}{4Dt} \right) dx = W \operatorname{erfc}\left(\frac{x}{\sqrt{4Dt}} \right) \tag{2}$$

where erfc is the error function. Now we derive the magnetization transferred from the water slab to our protein, assuming the protein has a fixed width *a* from the edge of the water source :

$$\mathrm{M}_{\mathrm{prot}}(t) = \int_{0}^{a} W \operatorname{erfc}\left(\frac{x}{\sqrt{4Dt}} \right) dx = \frac{2W\sqrt{Dt}}{\sqrt{\pi}} \left[1 - \exp\left(\frac{-a^2}{4Dt} \right) \right] + aW \operatorname{erfc}\left(\frac{a}{2\sqrt{Dt}} \right) \tag{3}$$

The above equation can be simplified using the parameter $b = \frac{a}{2\sqrt{Dt}}$:

$$\mathrm{M}_{\mathrm{prot}}(t) = 2W\sqrt{Dt} \left[\frac{1}{\sqrt{\pi}} \left(1 - e^{-b^2} \right) + 2b \operatorname{erfc}(b) \right] \tag{4}$$

At short mixing times where $2\sqrt{Dt} << a$, $b \to \infty$, thus equation (4) reduces to:

$$\mathrm{M}_{\mathrm{prot}}(t \to 0) \approx \frac{2W}{\sqrt{\pi}} \sqrt{Dt} \tag{5}$$

Thus, the initial-regime water-transferred magnetization is directly proportional to the water density W. At long mixing times where $2\sqrt{Dt} >> a$, $b \to 0$, and equation (4) reduces to:

$$\mathrm{M}_{\mathrm{prot}}(t \to \infty) \approx \frac{2W}{\sqrt{\pi}}\sqrt{Dt}b = \frac{Wa}{\sqrt{\pi}} \tag{6}$$

The intensity ratio S/S_0 between short and long mixing times is thus inversely related to the protein-water distance *a*:

$$\frac{S}{S_0} = \frac{\mathrm{M}_{\mathrm{prot}}(t \to 0)}{\mathrm{M}_{\mathrm{prot}}(t \to \infty)} \approx \frac{2\sqrt{Dt}}{a} \tag{7}$$

In addition, the water-transferred magnetization in the initial regime (eq. 5) is proportional to the amount of water, W, and the square root of the effective spin diffusion coefficient *D*. This means that the ratio of the initial-regime spectral intensities between the two samples reports the ratio of the products of the amount of water and the square root of the effective diffusion coefficient in the low-pH and high-pH channels. Since *D* is larger at high pH than low pH, the ratio of the initial-regime intensities represents an upper bound to the relative amount of water:

$$\frac{S_{\mathrm{pH4.5}}}{S_{\mathrm{pH7.5}}} \approx \sqrt{\frac{D_{\mathrm{pH4.5}}}{D_{\mathrm{pH7.5}}}}\frac{W_{\mathrm{pH4.5}}}{W_{\mathrm{pH7.5}}} \geq \frac{W_{\mathrm{pH4.5}}}{W_{\mathrm{pH7.5}}} \tag{8}$$

Water T_2' and $R_{1\rho}$ relaxation analysis

While the BPP theory can be applied readily to analysis of rapidly tumbling molecules in solution, accurate extraction of τ_{rot} in semi-solids is affected by coherent effects [6]. The longer τ_{rot} compared to bulk-like water correlation times (**Table S1**) indicates that the channel-bound water motion is highly restricted. The slower T_2' rates at low pH indicates that the channel water is more mobile in the low-pH channel on the nanosecond timescale.

The ^{1}H $R_{1\rho}$ relaxation dispersion profiles (**Fig. 3d**) can be described by the Bloch-McConnell formalism[7] to extract the kinetic parameters of exchange. The low-pH sample shows lower $R_{1\rho}$ values for channel-bound water at both low and high spin-lock fields (**Fig. 3d**). Fitting the dispersion data to a two-state exchange model (**Table S2**) yielded an $R_{1\rho}^0$ of 76±5 s^{-1} at high-pH and 39±4 s^{-1} at low-pH, and an exchange time τ_{ex} of 9.5±0.9 µs at high-pH and 5.2±0.3 µs at low-pH. For both samples, we obtain $\phi_{ex} = p_1p_2\Delta\omega^2 \sim 20\cdot10^6$ rad^2 s^{-2}, where p_1 and p_2 are the population fractions of the two states and $\Delta\omega$ is the isotropic shift difference between the two states. This chemical shift difference can be between protein-bound and protein-unbound water in the channel, or between bound water protons and labile protein protons. However, the exchange rates of $1/\tau_{ex} \sim 10^5$ s^{-1} are faster than the exchange rates of all labile OH and NH protons, except for histidine at ~273 K [8]. If the two populations are each ~50%, the ϕ_{ex} values indicate a chemical shift difference of ~1.4 kHz for the high-pH channel water and ~1.6 kHz for low-pH channel water. Conversely, if we assume the rarer state is 1% populated, then the ϕ_{ex} values indicate chemical shift differences of ~7 kHz for the high-pH sample and ~8 kHz for the low-pH sample. This ~10

ppm difference would be consistent with exchange between a water proton and a histidine sidechain proton, and the k_{ex} of 5.2±0.3 μs at low pH is consistent with the expected exchange rate between histidine and water at pH 4.5 [3], however at pH 7.5 we expect an exchange rate of 100,000 s^{-1} or more [9], which is an order of magnitude faster than the observed k_{ex} at pH 7.5. Therefore, based on the exchange rates fit to the $R_{1\rho}$ relaxation dispersion curves, we do not attribute the dispersion to exchange between histidine and water, but rather attribute it to exchange between two water populations. It is unlikely for the two pools of water that undergo fast exchanging to have large shift differences of ~10 ppm (which is ~8 kHz on the 800 MHz spectrometer). Therefore we assume the two states are both significantly populated and chemical shift differences of ~2 ppm. This chemical shift difference is much smaller than the exchange rate, which is consistent with the observation of a single water ^{1}H peak in the spectrum. Although 2 ppm is a large difference for two exchanging water populations, this isotropic chemical shift difference is similar to the 1.1 ppm difference between buried and hydration water interacting with ubiquitin encapsulated within a reverse micelle [10] and the 2.6 ppm difference in chemical shifts of water adsorbed to silica and alumina oxides [11].

In contrast to the ^{13}C-detected experiments, the ^{1}H-detected $R_{1\rho}$ experiments with a 40 ms T_2' filter to select for bulk-like water show no relaxation dispersion (**Fig. S3e**) and much slower $R_{1\rho}^0$ of 5.3±1.9 s^{-1} at high pH and 5.6±0.7 s^{-1} at low pH. When the ^{1}H-detected $R_{1\rho}$ experiments were conducted without a T_2' filter, which capture both bulk-like and lipid-bound water, then we observed a small dispersion that is similar for the high- and low-pH samples (**Fig. S3e**). The $R_{1\rho}^0$ values are 23±3 s^{-1} at high pH and 20±5 s^{-1} at low pH, while the τ_{ex} times are 3.6±0.8 μs at high pH and 3.0±0.9 μs at low pH. Precise interpretation of these microsecond exchange processes is complicated by the presence of coherent contributions under MAS, which can bias the extracted exchange parameters[6,12,13]. Nevertheless, the 2-10 fold faster $R_{1\rho}^0$ in the ^{13}C-detected experiments compared to the ^{1}H-detected experiments indicates that the motion of channel-bound water is significantly anisotropic. Moreover, the slower intrinsic relaxation rate and shorter exchange time constant for the low-pH sample indicate that the channel-bound water is more dynamic on the microsecond timescale in the open channel than in the closed channel.

Supplementary Table 1. ^{1}H-detected and ^{13}C-detected water ^{1}H T_2' relaxation times and the resulting rotational correlation times τ_{rot} calculated using the BPP theory [14].

Sample	Detection nucleus	Water Pool	T_2'	τ_{rot}
pH 7.5	^{1}H	Bulk-like	39.4±1.2 ms	0.68 ns
	^{1}H	Lipid	1.5±0.2 ms	20 ns
	^{13}C	Channel	4.0±0.1 ms	7.6 ns
pH 4.5	^{1}H	Bulk-like	47.7±2.2 ms	0.52 ns
	^{1}H	Lipid	2.2±0.3 ms	14 ns
	^{13}C	Channel	5.5±0.1 ms	5.6 ns

Supplementary Table 2. ^{1}H-detected and ^{13}C-detected water ^{1}H $R_{1\rho}$ relaxation dispersion fits assuming a two-state exchange process.

Sample	Detection nucleus	T_2' filter	Water Pool	$R_{1\rho}^0$	τ_{ex}	τ_{ex} (10^6 rad^{2}s^{-2})
pH 7.5	^{1}H	None	Bulk-like and Lipid	23±3 s^{-1}	3.6±0.8 μs	5±2
	^{1}H	40 ms	Bulk-like	5.3±1.9 s^{-1}	–	–
	^{13}C	None	Channel	76±5 s^{-1}	9.5±0.9 μs	19±2
pH 4.5	^{1}H	None	Bulk-like and Lipid	20±5 s^{-1}	3.0±0.9 μs	6±3
	^{1}H	40 ms	Bulk-like	5.6±0.7 s^{-1}	–	–
	^{13}C	None	Channel	39±4 s^{-1}	5.2±0.3 μs	26±2

Supplementary Table 3. Energy minimization and equilibration protocol and accompanying force constants with units of kJ mol^{-1} nm^{-2}.

Duration	Ensemble	BB restraint	SC restraint	LH restraint	LT restraint
5000 steps	EM	4000	2000	1000	1000
25 ps	NVT	4000	2000	1000	1000
25 ps	NVT	2000	1000	1000	400
25 ps	NPT	1000	500	400	200
100 ps	NPT	500	200	200	200
100 ps	NPT	200	50	40	100
100 ps	NPT	50	0	0	0
30 ns	NPT	0 or 50 (see methods)	0	0	0

Supplementary Table 4. Average channel-axis coordinates for Cα atom of residues I7 to H27, taken over the four independent unrestrained 100 ns MD simulations of the closed BM2 channel (+0/+1 H19/H27 charge state) and the open channel (+4/+4 H19/H27 charge state) at 277K, respectively.

Residue	$\langle Z\rangle_{closed}$ (Å)	$\langle Z\rangle_{open}$ (Å)
I7	-14	-13.5
L8	-12.2	-11.2
S9	-11.5	-10.9
I10	-10.1	-9.9
C11	-8.0	-7.4
S12	-6.7	-6.0
F13	-5.8	-5.6
I14	-3.9	-3.8
L15	-2.1	-1.6
S16	-1.2	-0.7
A17	0	0
L18	2.2	2.2
H19	3.6	4.0
F20	4.3	4.6
M21	6.0	5.8
A22	8.1	8.1
W23	9.1	9.4
T24	10.0	10.0
I25	11.8	11.9
G26	13.4	13.8
H27	14.8	14.7

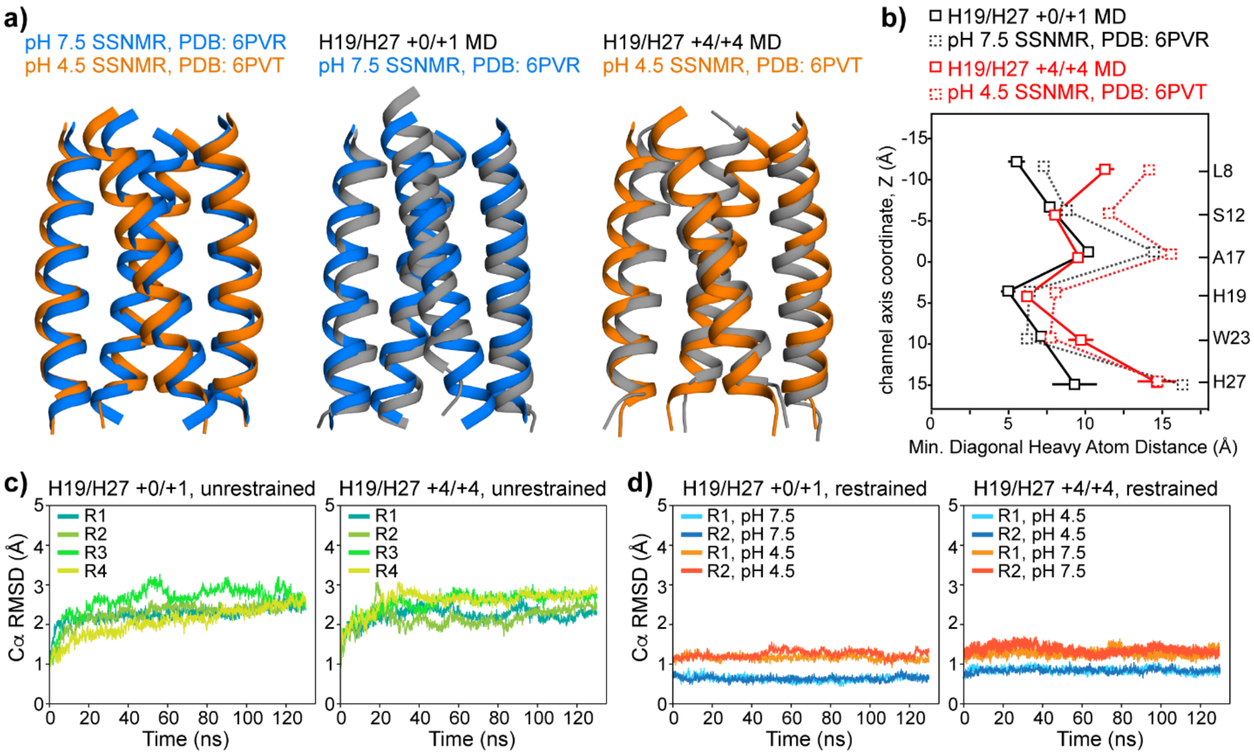

Supplementary Figure 1. Comparison of the MD and solid-state NMR structures of BM2 and the time evolution of MD trajectories. (**a**) (*left*) Overlay of the lowest energy state from the pH 7.5 (blue, PDB ID 6PVR) and pH 4.5 (orange, PDB ID 6PVT) solid-state NMR structural ensembles. (*right*) Overlays of the backbone traces of the MD equilibrated structures (grey) and the solid-state NMR experimental structures (red). (**b**) Minimum diagonal heavy-atom distances, a proxy for the pore radius, as a function of the channel-axis coordinate. The calculation was carried out for L8, S12, S16, H19, W23, and H27. There is good agreement between the MD equilibrated structures and the experimental solid-state NMR structures. (**c**) Time-evolution of Cα RMSD with respect to the solid-state NMR structures in the unrestrained MD trajectories. The four replicates (R1-R4) are shown in different shades of green. (**d**) Time-evolution of Cα RMSD with respect to the solid-state NMR structures in the restrained MD trajectories. The H19/H27 +0/+1 and +4/+4 MD states were restrained either to the pH 7.5 (PDB ID 6PVR) or the pH 4.5 (PDB ID 6PVT) solid-state NMR structures for two replicates. These restrained simulations show average Cα RMSDs below 1.50 Å.

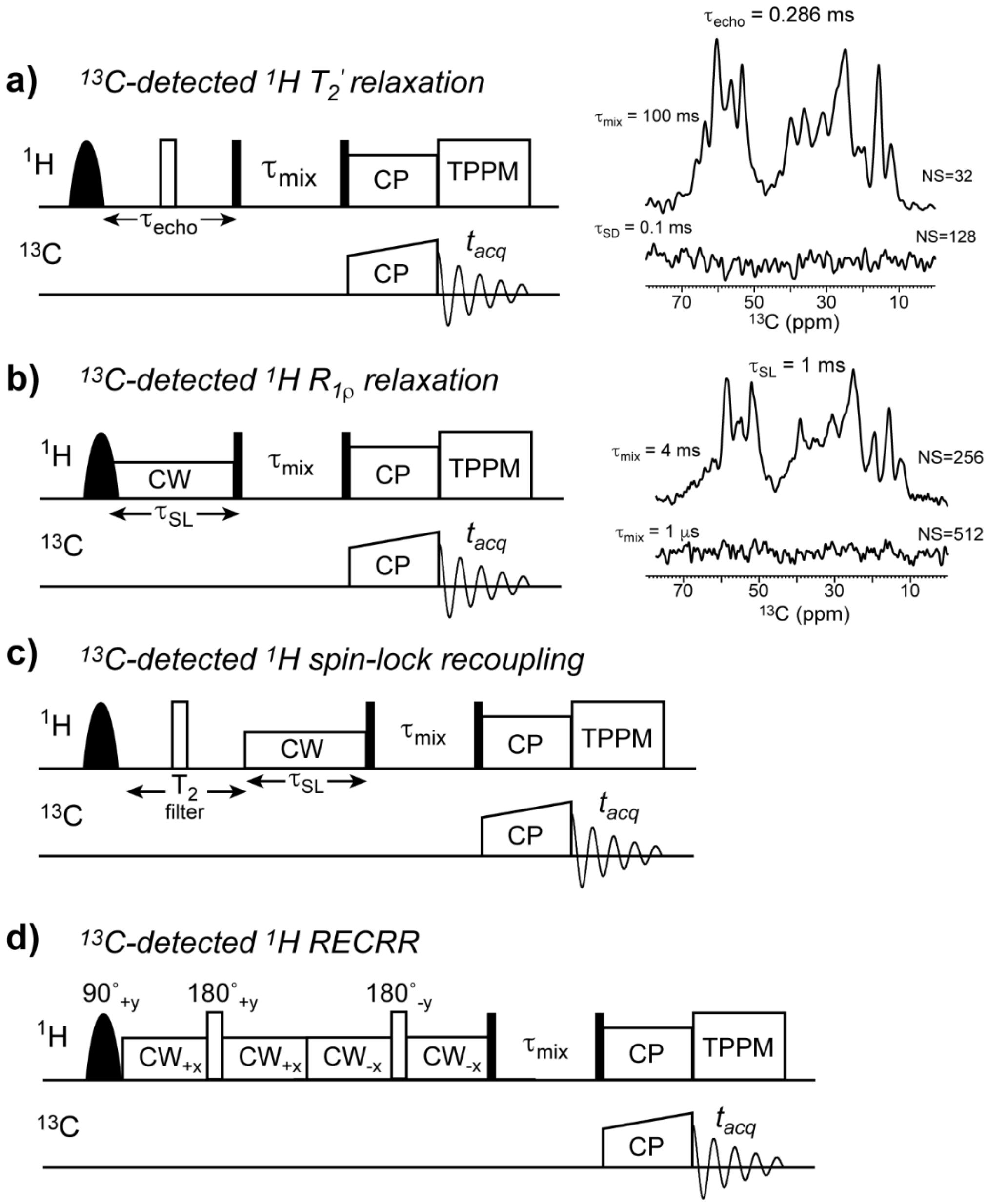

Supplementary Figure 2. Pulse sequences for the solid-state NMR experiments to detect water dynamics. (**a**) ^{13}C-detected ^{1}H T_2' experiment begins with a selective ^{1}H excitation pulse, followed by a variable T_2 filter period, a spin diffusion period τ_{mix}, and cross-polarization (CP) to transfer water ^{1}H polarization to protein ^{13}C for detection. Shown on the right is a pair of control spectra measured with a τ_{echo} period of 0.286 ms and with τ_{mix} of 100 ms and 0.1 ms. The 0.1 ms spectrum has no signal intensity, indicating that all protein ^{1}H magnetization is suppressed by the selective echo and a 0.286 ms echo period. Shown on the right is a pair of control spectra measured with 1 ms τ_{SL} and τ_{mix} times of 4 ms and 1 µs. The null intensities in the 1 µs spectrum indicate that all protein ^{1}H magnetization is suppressed. (**c**) ^{13}C-detected ^{1}H $R_{1\rho}$ spin-lock recoupling experiment. A ^{1}H T_2' filter precedes the spin-lock recoupling pulse to remove the magnetization of rigid protons. (**d**) ^{13}C-detected ^{1}H RECRR experiment.

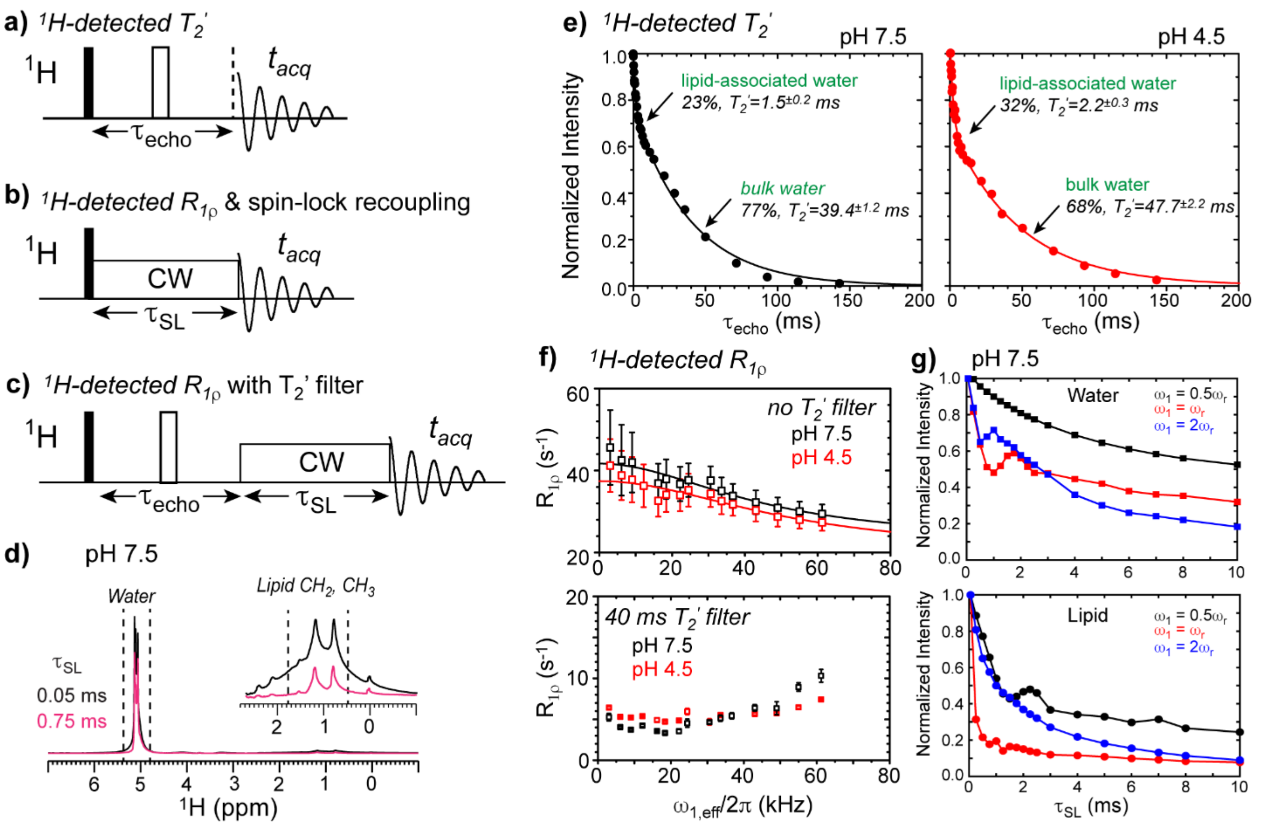

Supplementary Figure 3. ^{1}H-detected T_2' relaxation, $R_{1\rho}$ relaxation, and spin-lock recoupling of water in membrane-bound BM2 samples. All spectra were measured at a sample temperature of ~273 K. (**a**) Pulse sequence for measuring ^{1}H T_2'. (**b**) Pulse sequence for measuring ^{1}H $R_{1\rho}$ and for ^{1}H spin-lock recoupling. (**c**) Pulse sequence for measuring ^{1}H $R_{1\rho}$ that utilizes a T_2' filter to select for mobile, isotropic water. (**d**) Representative ^{1}H spectra at two spin-lock times for the pH 7.5 sample. The integration areas for the water peak and the lipid chain ^{1}H signals are indicated with dashed lines. (**e**) ^{1}H-detected water apparent transverse relaxation decays at pH 7.5 and pH 4.5. Both decays are biexponential. The small component of fast initial decay results from lipid-associated water while the major component of slow decay results from bulk-like water. The populations and T_2' values are indicated. (**f**) ^{1}H-detected on-resonance $R_{1\rho}$ relaxation dispersion curves as a function of spin-lock field strength. (*top*) The pH 7.5 (*black*) and pH 4.5 (*red*) samples both exhibit relaxation dispersion. (*bottom*) Addition of a 40 ms ^{1}H T_2' filter before the spin-lock period to suppress the lipid-associated water removed the relaxation dispersion, indicating that lipid-associated water is responsible for the dispersion. (**g**) ^{1}H-detected spin-lock recoupling data for all water protons and for lipid chain protons in the high-pH BM2 sample. The curves were measured at ω_1 values of $0.5\omega_r$, ω_r, and $2\omega_r$. The water signal, which results from bulk-like, lipid-associated and channel water, shows oscillations at $\omega_1 = \omega_r$ and $2\omega_r$ but not at $\omega_1 = 0.5\omega_r$, indicating CSA recoupling. The minima of the oscillations are higher than the minima of channel-bound water (**Fig. 5a**), indicating that only a fraction of all water is anisotropic. The lipid protons (bottom panel) show oscillations at $\omega_1 = 0.5\omega_r$ and ω_r but little oscillation at $\omega_1 = 2\omega_r$, indicating that ^{1}H-^{1}H dipolar interaction is recouped.

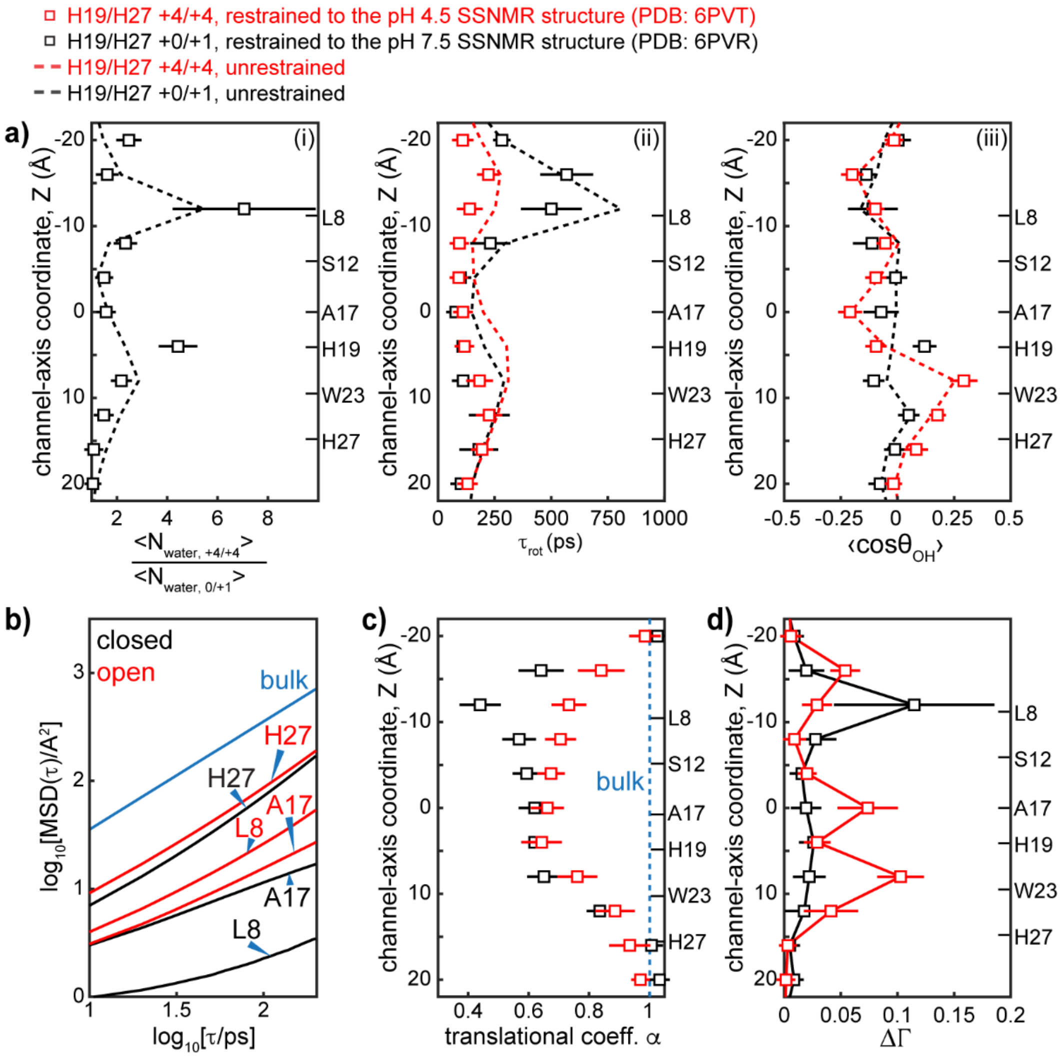

Supplementary Figure 4. (**a**) Comparison of unrestrained and restrained MD simulations of channel water dynamics. Simulations for channels whose backbone has been restrained to the solid-state NMR structures are shown as squares while unrestrained simulations are shown as dashed lines. Results are insensitive to the use of position restraints. Shown are: (**i**) the ratio of the water counts between the open and closed BM2 channels, (**ii**) the rotational correlation time, τ_{rot}, and (**iii**) time-averaged $\cos\theta_{OH}$ of channel water. (**b**) Mean-squared displacement of water as a function of time near various pore-lining residues for the closed (*black*) and open (*red*) channels. Bulk-like water diffusion is plotted as a blue line for comparison. Diffusion is faster in the open, H19/H27 +4/+4 channel on average. In both the open$^{+4/+4}$ channel and the closed$^{0/+1}$ channel, diffusion is the fastest near H27 (Z=20). In the closed$^{0/+1}$ state, diffusion is especially slow near L8 (Z=-12). (**c**) Translational diffusion power-law exponent α along the channel axis. All α values inside the channel are below 1, indicating sub-diffusive behavior. (**d**) Entropy of the $P(\cos(\theta_{OH}))$ distribution, which reports water orientations independent of the reference vector direction. A higher value means more ordered water. The open channel has a larger proportion of ordered water than the closed channel.

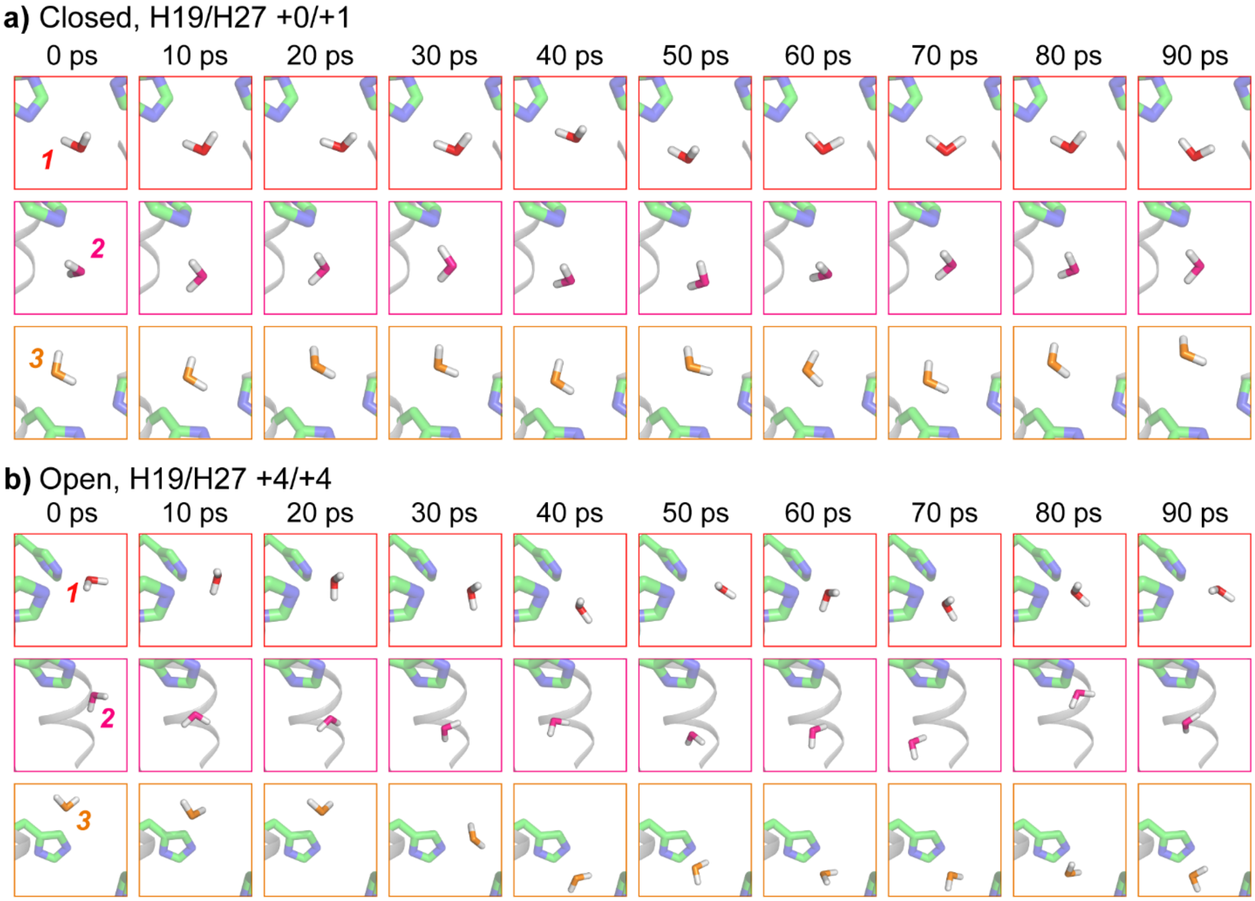

Supplementary Figure 5. Snapshots of representative water molecules near H19 over a 100 ps interval. **(a)** Closed channel represented by +0/+1 protonation for the H19/H27 tetrads. **(b)** Open channel represented by +4/+4 protonation for the H19/H27 tetrads.

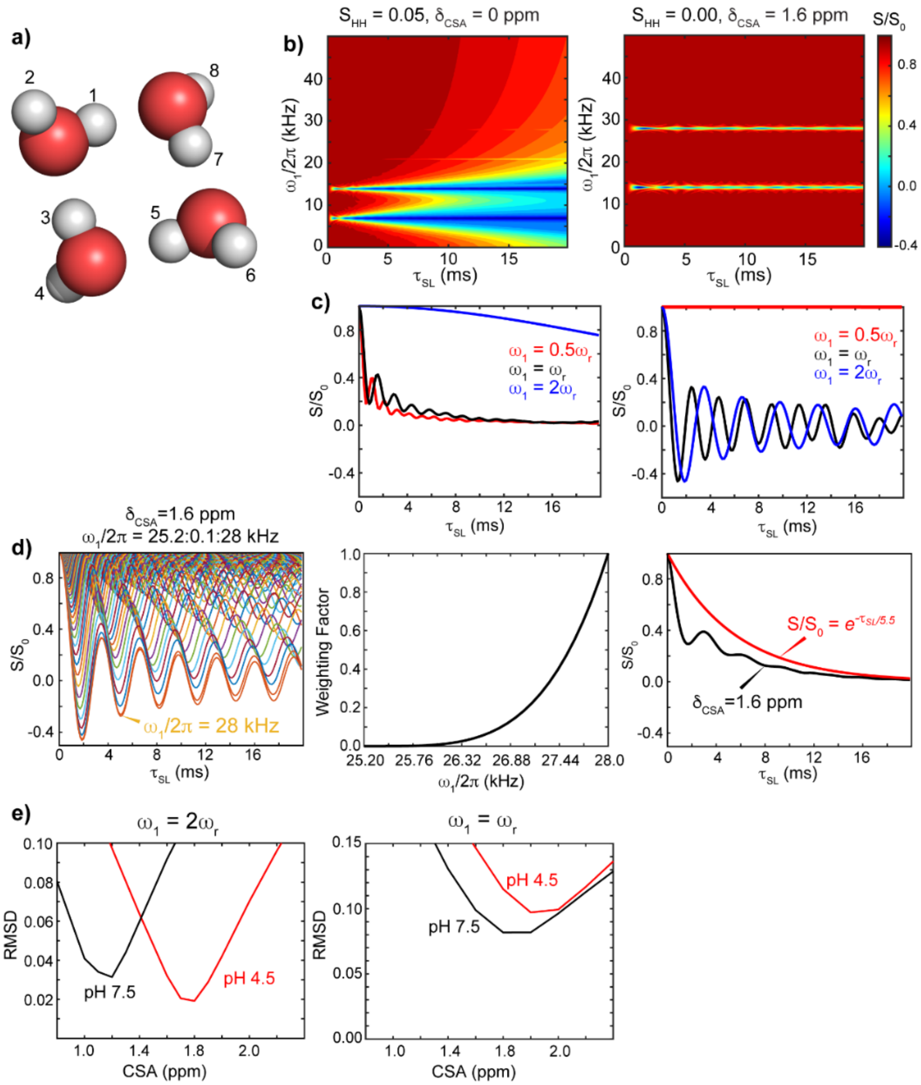

Supplementary Figure 6. Numerical simulations and RMSD plots of the ^{1}H spin-lock recoupling experiment for extracting motionally averaged water ^{1}H CSA. (**a**) The spin system used in the simulations. The coordinates of eight protons in four water molecules were included. All ^{1}H spins were on resonance with the rf carrier frequency. All simulations were done for 14 kHz MAS on an 800 MHz spectrometer. Experiments began with *x*-magnetization and the amount of magnetization remaining on spin #1 was monitored as a function of spin-lock time. The water molecules are symmetric such that spins #1, #3, #5, and #7 are in the same magnetic environment and spins #2, #4, #6, and #8 are in the same magnetic environment. (**b**) (*left*) 2D dephasing map where ^{1}H-^{1}H dipolar couplings were included with an order parameter S_{HH} = 0.05 but CSA was neglected. The spin-lock mixing time, τ_{SL}, is plotted on the *x*-axis, and the spin-lock field strength ω_1 is shown on the *y*-axis. There are two broad matching conditions at ω_1=0.5ω_r and ω_r. (*right*) 2D dephasing map in which a 1.6 ppm ^{1}H CSA was included for all protons, but ^{1}H-^{1}H dipolar couplings were neglected. There are two sharp matching conditions at ω_1=ω_r and 2ω_r. (**c**) Cross sections corresponding to spin-lock field strengths of ω_1=0.5ω_r, ω_r and_2ω_r from ^{1}H-^{1}H dipolar coupling (*left*) and ^{1}H CSA (*right*) dephasing maps. Note that the ^{1}H-^{1}H dipolar couplings never

cause the signal to decay below zero. We note that the simulations in (**b**) and (**c**) depict the unrealistic condition of having no rf inhomogeneity, however they serve as an illustrative map of what rotary resonance matching conditions result in coherent recoupling for dipolar couplings and CSA. (**d**) (*left*) 50 recoupling curves simulated for the $\omega_1=2\omega_r$ condition plotted on one *xy*-plane to show the dispersion in the curve shapes and depths. Curves were simulated for rf field strengths corresponding to 90-100% of 28 kHz in 0.36% increments. (*middle*) Normalized x^4 weighting function that accounts for most of the sample experiencing the desired 28 kHz rf field strength, with only a miniscule fraction of the sample subjected to the 0.9*28 kHz inhomogeneity. (*right*) Exponential decay curve with a 5.5 ms relaxation time (*red*) to account for relaxation during the experiment. Final CSA recoupling curve for the $\omega_1 = 2\omega_r$ matching condition with a 1.6 ppm ^{1}H CSA (*black*) that accounts for rf inhomogeneity and relaxation. (**e**) Root-mean-square deviation (RMSD) between our experimental and simulated recoupling curves. We note that these fits used a 5.0 ms exponential relaxation time because this lead to the lowest RMSD's for both samples.

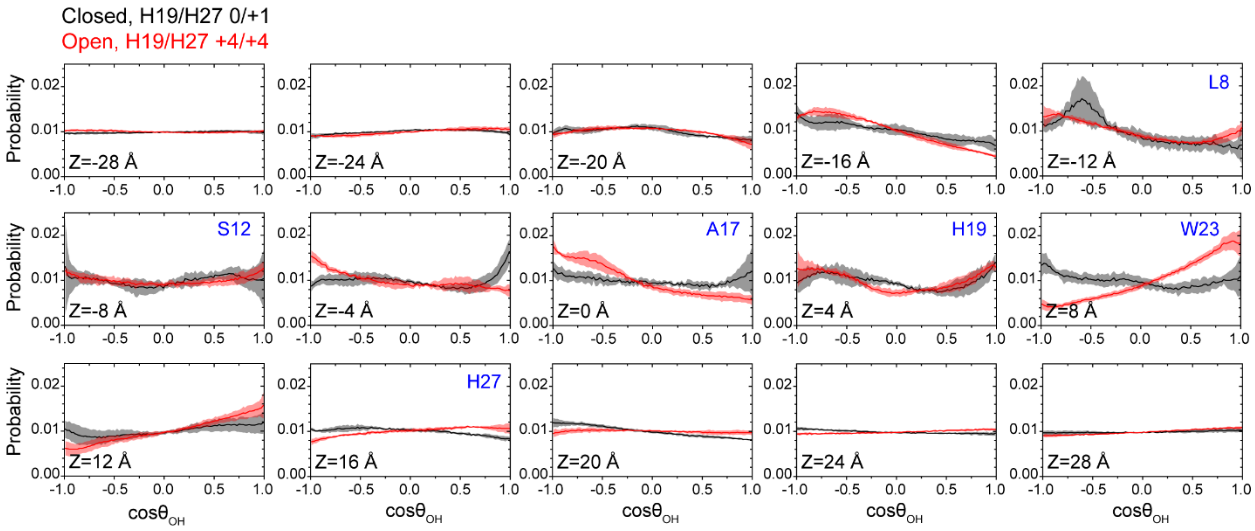

Supplementary Figure 7. Probability distributions of water order parameter cosθ$_{CH}$ along the channel axis in 4 Å bins. Lines and shaded areas show the mean and standard deviation, respectively, across the four replicates.

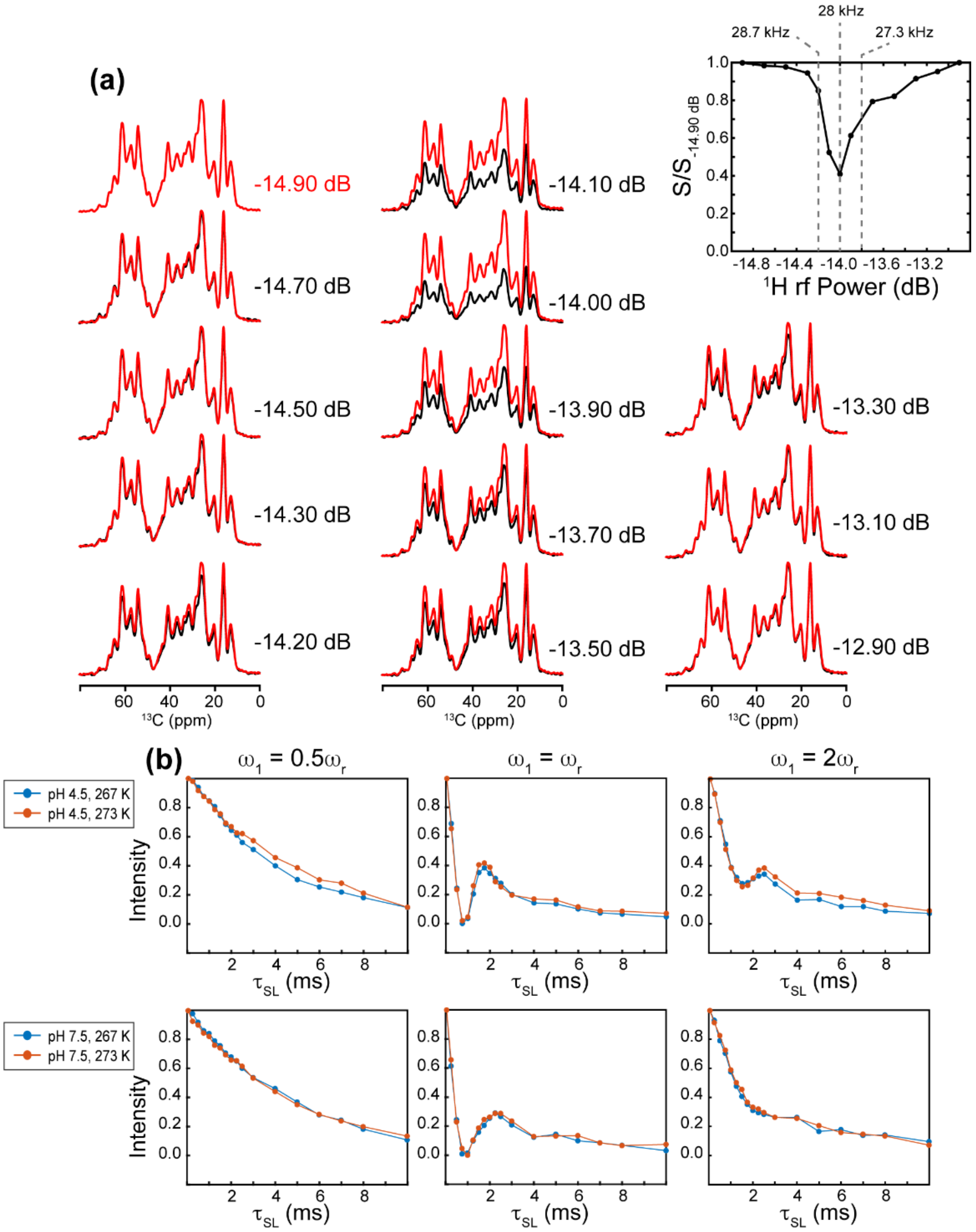

Supplementary Figure 8. (**a**) Experimental optimization of the ^{1}H spin-lock power for the $2\omega_r$ recoupling condition for the pH 4.5 sample. To optimize the rf power level, a minimum in the aliphatic intensity was found for a spin-lock recoupling time of 1 ms. This power level was optimized to within <0.1 dB, which would correspond to a 1.2% error in the applied rf power. (*upper right*) Integrated aliphatic intensity for the ^{1}H rf powers sampled in this optimization. The integrated intensity monotonically increases as the rf power is moved away from the optimal value. The same optimization routine was used to optimize the $2\omega_r$ matching condition for the pH 7.5 sample along with the $0.5\omega_r$ and ω_r matching conditions for both the pH 4.5 and pH 7.5 samples. (**b**) Comparison of ^{13}C-detected ^{1}H spin-lock recoupling experiments at sample temperatures of 273 K (**Fig. 5a**) and 267 K. We note that even though rotational motion and chemical exchange are expected to be slower at 267 K, we observe nearly identical recoupling curves.

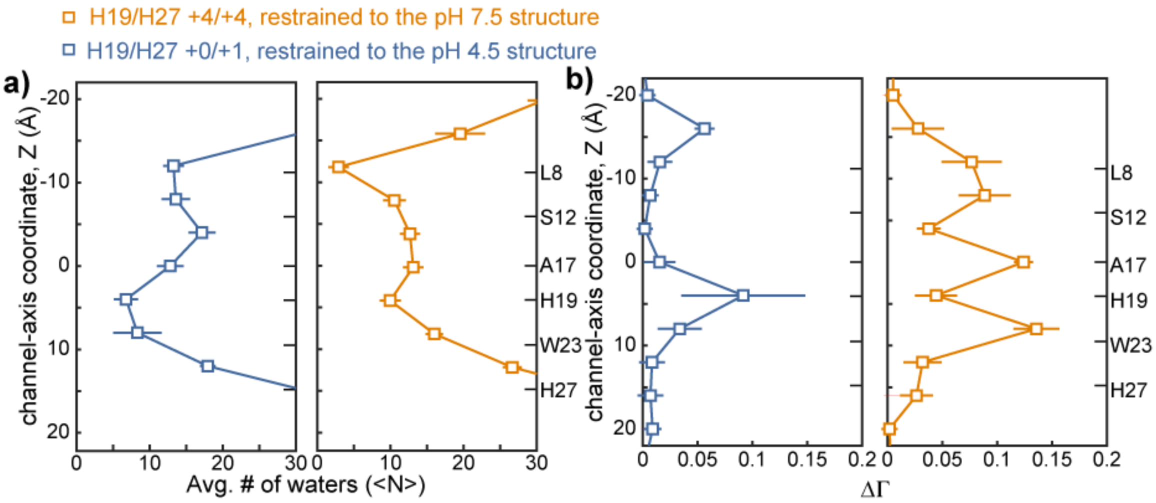

Supplementary Figure 9. Water simulations using +4/+4 charged H19/H27 tetrads restrained to the pH 7.5 solid-state NMR structure (PDB: 6PVR), and 0/+1 charged H19/H27 tetrads restrained to the pH 4.5 solid-state NMR structure (PDB: 6PVT). These unphysical conditions probe the relative effects of H19 charge and protein backbone conformation on channel-water dynamics. (**a**) Average number of water molecules in the channel. The +4/+4 state shows a large number of water molecules at the C-terminal H19-W23 juncture, even though the pH 7.5 structure with a tighter four-helix bundle is used for the simulations. (**b**) Entropy of $P(\cos(\theta_{OH}))$ distribution of channel water. The +4/4 state shows larger $\Delta\Gamma$, indicating larger water order.

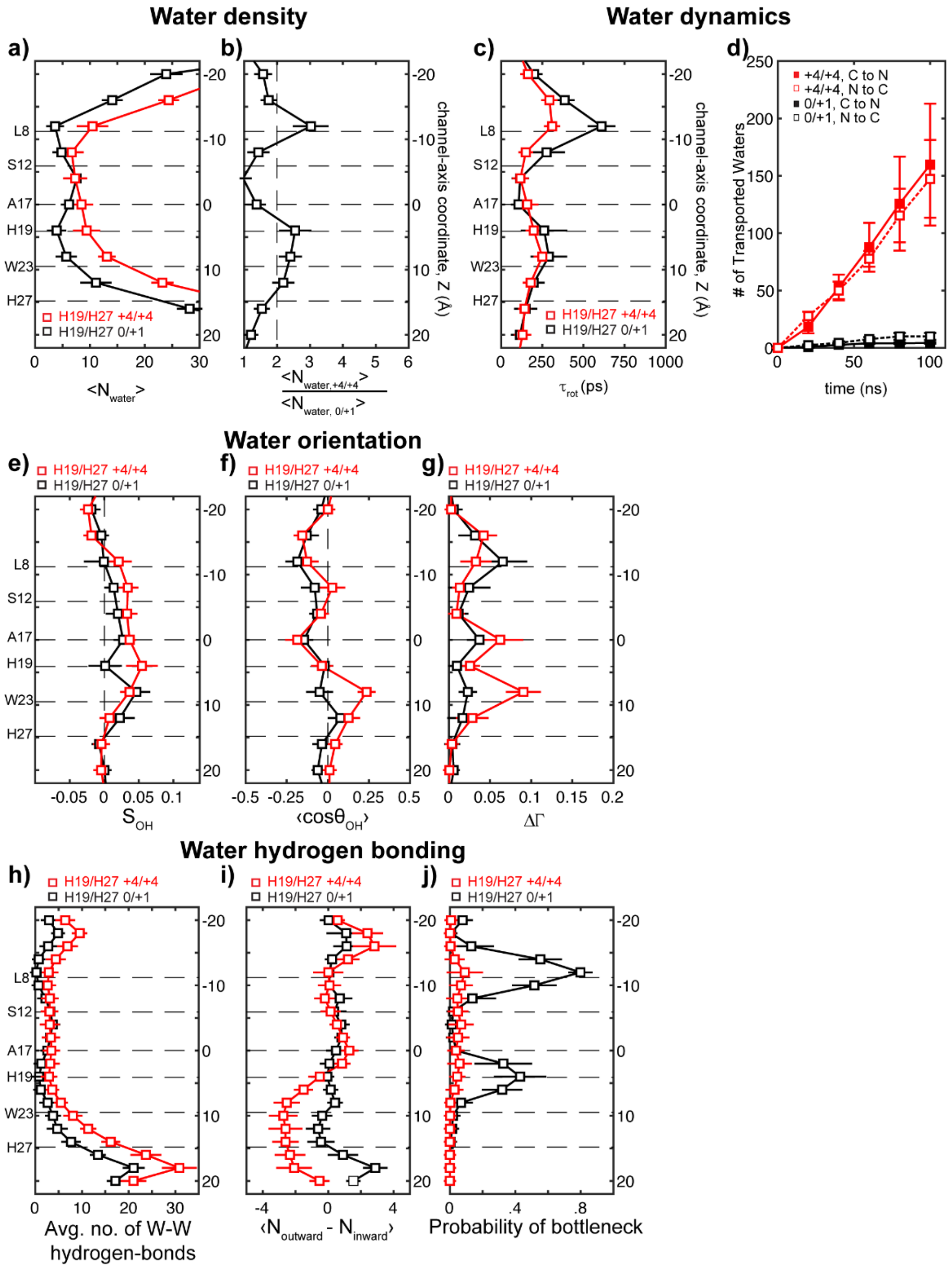

Supplementary Figure 10. Analysis of MD simulations performed at 297 K yield the same conclusions as the simulations at 277 K, presented in the main text. (**a**) Number of water molecules in the BM2 pore, for comparison with **Figure 2d**. (**b**) The ratio of water molecules in the +4/+4 state compared to the 0/+1 state, vertical guideline shows a ratio of 2 fold, for comparison with **Figure 2e**. The +4/+4 state has ~2 fold more water as compared to the 0/+1 state, in agreement with the result at 277K. (**c**) The rotational auto-correlation time, for comparison with **Figure 3f**. In agreement with the result at 277K, rotational dynamics are faster in the +4/+4 state (average τ_{rot} is 196±4 ps) as compared to the 0/+1 state (average τ_{rot} is 214±16 ps). As expected, rotational motion is found to be slightly faster overall at 297K as compared to 277K. (**d**) The number of

water molecules transported across the BM2 channel as a function of time, for comparison with **Figure 3g**. In agreement with the result at 277K, more water molecules are transported in the +4/+4 state (1.54±0.44 water/ns) as compared to the 0/+1 state (0.07±0.02 water/ns). As expected, transport is enhanced at 297K as compared to 277K. (**e**) OH-bond NMR order parameter, as determined from the MD trajectories, for comparison with **Figure 5c**. (**f**) The expectation value of the cosine of the OH-bond angle, for comparison with **Figure 5e**. In agreement with the result at 277K, a change in the orientational preference is observed around H19 in the +4/+4 state, but not in the 0/+1 state. (**g**) The entropy in the $P(\cos(\theta_{OH}))$ distribution, quantified using the parameter $\Delta\Gamma$ (see methods), for comparison with **Figure S4d**. In agreement with the result at 277K, water is more ordered in the +4/+4 state as compared to the 0/+1 state. (**h**) The average count of water-water hydrogen bonds is shown, for comparison with **Figure 7b**. In agreement with the 277 K result, the average hydrogen bond count is similar or larger along most of the channel for the the +4/+4 state state than for the 0/+1 state. (**i**) The average directionality metric is shown, for comparison to **Figure 7d**. In agreement with the 277 K result, there is more directionality in the +4/+4 state, with a shift in directionality at H19. (**j**) The bottleneck probability is shown, for comparison with **Figure 7c**. As for the 277 K simulations, bottlenecks are more more likely for the 0/+1 state than for the +4/+4 state.

Supplementary References

1. Pattle, R.E. Diffusion from an Instantaneous Point Source with a Concentration-Dependent Coefficient. *Q. J. Mech. Appl. Math.* **12**, 407-409 (1959).
2. Ader, C. et al. Structural Rearrangements of Membrane Proteins Probed by Water-Edited Solid-State NMR Spectroscopy. *J. Am. Chem. Soc.* **131**, 170-176 (2009).
3. Sehgal, A.A., Duma, L., Bodenhausen, G. & Pelupessy, P. Fast proton exchange in histidine: measurement of rate constants through indirect detection by NMR spectroscopy. *Chem. Eur. J.* **20**, 6332-6338 (2014).
4. Kateb, F., Pelupessy, P. & Bodenhausen, G. Measuring fast hydrogen exchange rates by NMR spectroscopy. *J. Magn. Resn.* **184**, 108-113 (2007).
5. Liepinsh, E., Otting, G. & Wüthrich, K. NMR spectroscopy of hydroxyl protons in aqueous solutions of peptides and proteins. *J. Biomol. NMR* **2**, 447-465 (1992).
6. Schanda, P. & Ernst, M. Studying Dynamics by Magic-Angle Spinning Solid-State NMR Spectroscopy: Principles and Applications to Biomolecules. *Prog. Nucl. Magn. Reson. Spectrosc.* **96**, 1-46 (2016).
7. Palmer, A.G. & Massi, F. Characterization of the Dynamics of Biomacromolecules Using Rotating-Frame Spin Relaxation NMR Spectroscopy. *Chem. Rev.* **106**, 1700-1719 (2006).
8. Liepinsh, E. & Otting, G. Proton exchange rates from amino acid side chains—implications for image contrast. *Mag. Reson. Med.* **35**, 30-42 (1996).
9. Hu, F., Schmidt-Rohr, K. & Hong, M. NMR detection of pH-dependent histidine-water proton exchange reveals the conduction mechanism of a transmembrane proton channel. *J. Am. Chem. Soc.* **134**, 3703-3713 (2012).
10. Nucci, N.V., Pometun, M.S. & Wand, A.J. Site-resolved measurement of water-protein interactions by solution NMR. *Nat. Struct. Mol. Biol.* **18**, 245-249 (2011).
11. Gun'ko, V.M. & Turov, V.V. Structure of Hydrogen Bonds and 1H NMR Spectra of Water at the Interface of Oxides. *Langmuir* **15**, 6405-6415 (1999).
12. Smith, A.A., Ernst, M. & Meier, B.H. Because the Light is Better Here: Correlation-Time Analysis by NMR Spectroscopy. *Angew. Chemie. Int. Ed.* **56**, 13590-13595 (2017).
13. Smith, A.A., Ernst, M. & Meier, B.H. Optimized "detectors" for dynamics analysis in solid-state NMR. *J. Chem. Phys.* **148**, 045104 (2018).
14. Bloembergen, N., Purcell, E.M. & Pound, R.V. Relaxation Effects in Nuclear Magnetic Resonance Absorption. *Phys. Rev.* **73**, 679-712 (1948).
