# Supplementary material for: Water orientation and dynamics in the closed and open influenza B virus M2 proton channels
Source: Commun Biol. 2021 Mar 12;4:338. doi: 10.1038/s42003-021-01847-2 (PMC7955094; doi:10.1038/s42003-021-01847-2)
Supplement: Supplementary file 5 — Description of Additional Supplementary Files [file 42003_2021_1847_MOESM5_ESM.pdf]

Description of Additional Supplementary Files

File name: Supplementary Movie 1

Description: Movie showing 5 ns of MD trajectories for both the closed, H19/H27 0/+1 channel and the open, H19/H27 channel. These movies begin at 120 ns in replicate 1, which includes 30 ns of equilibration time. Water in the +4/+4 channel generally has faster rotational and translational motion than water in the 0/+1 channel. We note that for both charge states, water motion in the channel is highly retarded compared to bulk-like water, as expected for a confined channel. Water molecules within 4 Å of the BM2 transmembrane domain (Q6-N29) are shown. The L8 sidechain is highlighted to point out the clear bottlenecks that occur at this location in the 0/+1 channel, but not the +4/+4 channel, while H19 is highlighted to show the enhanced orientation preference of water molecules near the highly charged His tetrad in the +4/+4 channel.

File name: Supplementary Data 1

Description: Source data underlying plots shown in figures.
